# Implementing recovery resources in trauma care: impact and implications

**DOI:** 10.1097/OI9.0000000000000045

**Published:** 2019-11-22

**Authors:** Natasha M. Simske, Mary A. Breslin, Sarah B. Hendrickson, Kenneth P. York, Heather A. Vallier

**Affiliations:** MetroHealth Medical Center, Cleveland, Ohio, affiliated with Case Western Reserve University, Cleveland, Ohio

**Keywords:** recovery, satisfaction, self-efficacy, survivor, Trauma Survivor Network, TSN

## Abstract

**Objectives:**

To identify resources that patients perceive as helpful to their recovery and to characterize the impact of the Trauma Survivor Network (TSN), a program committed to enhancing recovery through education and engagement.

**Design:**

Prospective cohort study.

**Setting:**

Level 1 trauma center.

**Patients/Participants:**

Four hundred eighty-five patients with musculoskeletal injuries. Two hundred eleven were exposed to TSN resources (Group 1). One hundred thirty-five patients were treated during the same period with no exposure (Group 2, control). One hundred thirty-nine patients were treated 1 to 3 months prior to TSN implementation (Group 3, control).

**Intervention:**

TSN resources including educational materials, family classes, peer survivor visits, coaching, online services, and support groups.

**Main outcome measure:**

A survey to assess hospital experience and perceptions about recovery.

**Results:**

On a Likert scale from 0 to 5, patients were highly satisfied (mean 4.24), with no differences based on TSN exposure. Patients exposed to TSN programming reported greater perceived likelihood of recovery: mean 3.73 vs 3.41 vs 3.38, Group 1 vs Group 2 vs Group 3 (*P* = .05) and regarding return to daily activities: 3.69 vs 3.49 vs 3.10, *P* = .003. Fifty-three percent of Group 1 patients exposed to TSN programming utilized peer relationships and 42% read the educational materials provided. Support groups were also popular, with 26% of patients attending at least 1 session. Patients who recalled utilization of TSN services were overall highly satisfied with these services, mean 4.42.

**Conclusion:**

Patients were overall highly satisfied with their hospital stay, with those exposed to TSN services reporting greater perceived likelihood of recovery and return to daily activities. Development of nontraditional services, including peer visitation and support groups, appears to enhance expectations about recovery.

## 1 Introduction

Sequelae of trauma, including physical, social, and psychological elements, often persist long after patients have been discharged from the hospital. This can pertain to both substantial physical disabilities, as well as psychiatric illnesses that exacerbate these injuries in up to 45% of trauma patients.^[1–7]^ Mental illness may slow recovery and potentially hinder satisfactory outcomes. Psychiatric illnesses have been associated with poor adherence to treatment recommendations, higher rates of complications, and greater risk for subsequent intentional and unintentional injury recidivism.^[2,4,6–10]^ Patients with mental illness are also at risk for poor engagement, another factor possibly contributing to suboptimal recovery following orthopaedic trauma.^[11,12]^

The TSN was founded to address these concerns by improving engagement, increasing support, and creating a community of survivors. TSN was originally developed through collaboration with the American Trauma Society (ATS) researchers and clinicians to address the psychosocial traits surrounding care after traumatic injury that are often left unaddressed.^[13]^ TSN programming is based on 4 key tenets, including self-management, peer support, access to information, and online networking. These tenets are based on evidence from their use in nontrauma patient care, where functional outcomes have purportedly improved as a result.^[13–18]^

The aim of the present study is to evaluate patient satisfaction with TSN services and the impact of these services on patient perceptions about recovery. It is hypothesized that patients interacting with TSN will have greater satisfaction, optimism, and self-efficacy, indicating a constructive impact of this program and benefits of future widespread application.

## 2 Methods

### 2.1 The trauma survivor network

The development of resources for trauma patients and their families, known as Trauma Recovery Services at this level 1 trauma center, began in 2013. The effort was initiated through a study funded by the Department of Defense (DOD), facilitated by the Major Extremity Trauma Research Consortium (METRC). The goal of that study was to implement TSN programming, including elements of peer mentorship, support groups, and individualized counseling, under the direction of a Trauma Recovery Coach. Accordingly, the Trauma Collaborative Care (TCC) Program was a multicenter study designed to improve outcomes following high-energy orthopaedic trauma. This was accomplished through introduction of patients at 6 intervention sites to these same TSN services.^[14]^ Preliminary data indicate that implementing programs such as these is beneficial to both patients and surgeons.^[19]^ Data collection for this study took place prior to the onset of the METRC TCC study.

### 2.2 Control vs experimental groups

The patient population consisted of adult patients admitted to an urban level 1 trauma center for musculoskeletal injuries. Patients with traumatic brain injury, baseline dementia, or spinal cord injury were excluded from the study, as were patients with chest or abdomen injuries of Abbreviated Injury Scale (AIS) >2. Patients were divided into an intervention group and 2 control groups, and were matched for similar age, sex, mechanism of injury, and fracture type. Fracture types were divided into 5 groups: upper extremity, pelvis, acetabulum, femur, and tibia or ankle. Six patients with below-knee amputations were placed into a separate category and similarly matched across groups. The experimental group consisted of patients exposed to TSN services by a Trauma Recovery Coach over a 5-month period from May through September 2013 (Group 1). Two hundred eleven patients (43.5%) comprised Group 1, admitted for musculoskeletal injuries and received TSN services. The first control group consisted of patients who were not exposed to TSN services at the hospital during the same time period (Group 2: N = 135; 27.8%). Another control group consisted of patients who were treated at the same institution between 1 to 3 months prior to the initiation of the TSN programming and therefore had no exposure to TSN services (Group 3: N = 139; 28.6%). This additional control group was delineated to control for potential provider or institutional differences that accompanied TSN establishment.

### 2.3 Patient surveys

**Figure 1:**
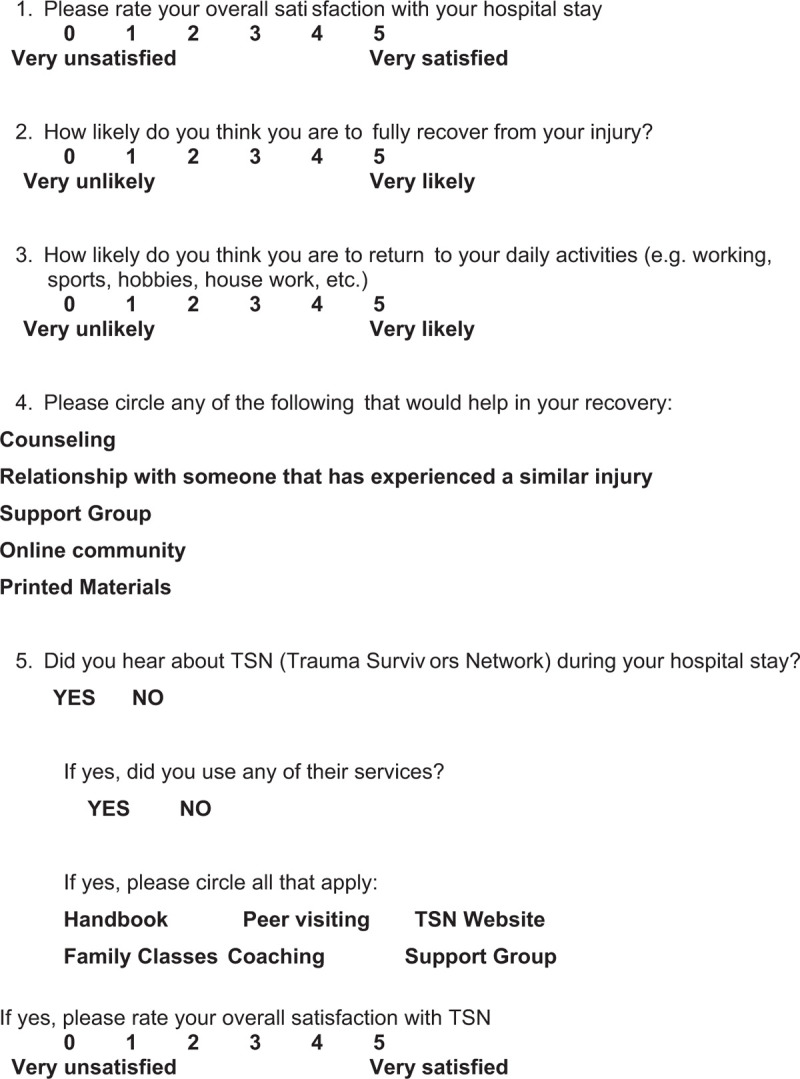
Survey sent to all patients.

Surveys were sent via mail to all patients from each of the groups (Fig. 1). The survey consisted of 5 questions developed by our research team, and without validation. If there was no response a second mailed survey was sent. Phone contact was made by a trained member of the research team if there was no response to mailed surveys.

### 2.4 Statistical analysis

Demographics including age, sex, mechanism of injury, and fracture characteristics were obtained using hospital electronic medical records. Control groups consisted of patients obtained from the trauma registry and were matched using demographic and injury variables. One-way ANOVA tests were used to compare ratings of satisfaction and self-efficacy between patients in the 3 experimental groups. Pearson chi-squared tests were employed for categorical variable comparisons. In all cases, *P* < .05 was considered to represent a significant difference.

## 3 Results

### 3.1 Response rate

Four hundred eighty-five surveys were sent to patients, with 160 responses for an overall response rate of 32.9%. Group 1 had the highest response rate, 35.5%, with 75 of 211 sent surveys being returned. Group 2 had the second highest, 34.1%, with 46 of 135 patients returning surveys. Group 3 had the lowest response rate, 28.1%, with only 39 of 139 patients responding to mailed surveys. Response rates were no different between groups (*P* = .57).

### 3.2 Demographics

The mean age of patients who received surveys was 43.0 years (SD = 18.1), and 65.8% were male. The most common mechanism of injury was a motor-vehicle collision (MVC), (n = 155, 32%), followed by falls (n = 154, 31.8%). The most prevalent injuries were to the tibia or ankle (n = 148, 30.5%) and to the femur (n = 129, 26.6%). Overall, patients were well matched. Survey groups were only dissimilar in terms of motorcycle collisions (MCCs), pedestrian collisions, and upper extremity injuries. Group 1, the TSN-exposed cohort, had substantially more operative upper extremity fractures (19%, *P* = .0011). All other demographic and injury variables did not reach statistical significance (Table 1).

**Table 1:**
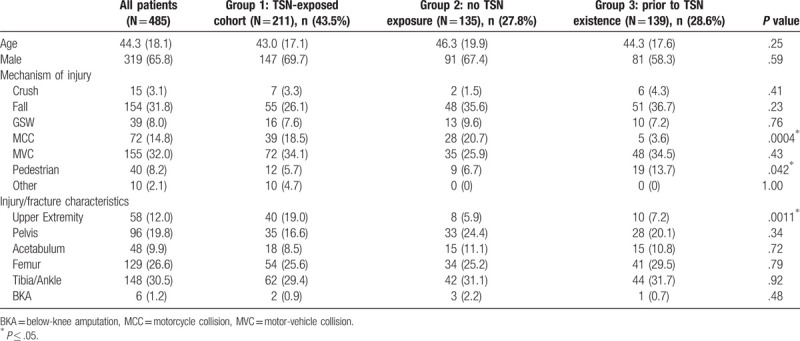
Demographic analysis of Group 1, and control Groups 2 and 3.

### 3.3 Satisfaction with hospital stay

On average, patients were highly satisfied with their hospital experience, with an average rating of 4.24 (SD = 1.10) across all groups. Patient groups reported similar satisfaction rates: Group 1: 4.16, Group 2: 4.39, and Group 3: 4.17. There were no significant differences between control and experimental groups in terms of patient satisfaction with his or her hospital stay (Table 2).

**Table 2:**
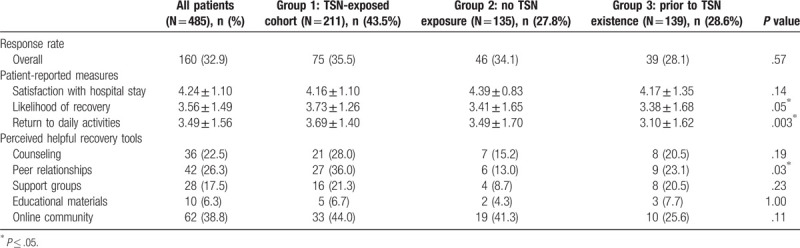
Survey responses by intervention versus control groups 2 and 3.

### 3.4 Self-efficacy

Overall, patients had lower self-efficacy scores compared to satisfaction ratings. Average rating of likelihood of recovery was 3.56 (SD = 1.49) and mean rating for likelihood of returning to daily activities was 3.49 (SD = 1.56) (Table 2). Patients exposed to TSN programming consistently rated a higher likelihood of recovery, 3.73 vs 3.41 for control Group 2 and 3.38 for control Group 3 (*P* = .05). Patients with TSN exposure also perceived a greater likelihood of return to daily activities. Group 1 reported an average score of 3.69 (SD = 1.4), compared to 3.10 (SD = 1.62) for Group 2 and 3.49 (SD = 1.70) for Group 3, *P* = .003.

### 3.5 Helpful recovery tools

All groups were asked to identify resources, from a list of 5 options, which would benefit them along their journey to recovery. One hundred sixty patients reported a total of 178 resources that they perceived would be helpful, with an average of 1.1 resources per respondent. An online community was most popular, with 62 respondents (38.8%) indicating that this may aid their recovery process. Peer relationships were also highly regarded, with 42 respondents (26.3%) indicating that this would be beneficial. Counseling services were also perceived as being a helpful recovery tool. Full results and breakdown according the group number are shown in Table 2.

### 3.6 Utilized TSN resources

Forty-one of the 75 Group 1 respondents (55%) answered the question about utilizing TSN resources. Recall was limited given that of these 41 individuals, only 19 patients (46.3%) indicated that they did use services, with an average of 1.5 per responding patient. Peer trauma survivor visitation was reported to be the most-used service with 52.6% of patients being visited by a peer or engaging in a peer relationship. The TSN handbook was also popular, with 42.1% reporting utilization of this resource.

**Figure 2:**
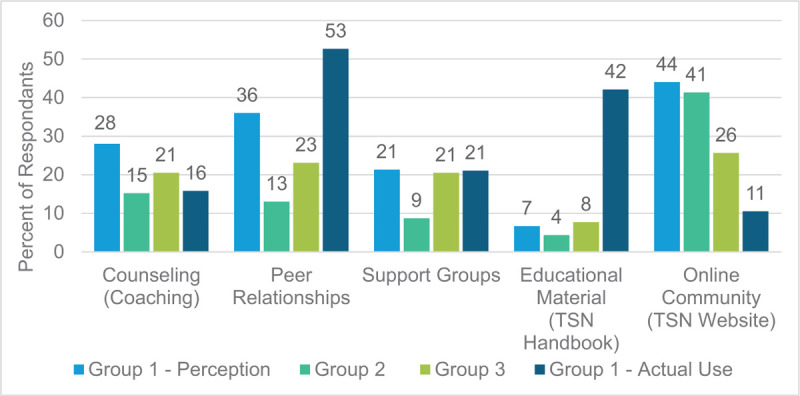
Group 1, 2, and 3's perception of helpful recovery resources compared to actual use of available services by Group 1. Notably, groups 2 and 3 served as control groups for patients without TSN exposure and treated pre- and post-TSN existence at our hospital.

Perception of beneficial services to recovery (assessed for all groups) did not always match the resources used by the intervention group (Fig. 2). Group 1 used fewer counseling/coaching resources (15.8% vs 22.5%, NS) and less web-based tools (10.5% vs 38.8%, *P* = .074) (Table 3). Patients exposed to TSN programming were more likely to use educational tools, compared to prior perceptions (42.1% vs 6.3%, *P* = .0008). Patients introduced to the TSN were also more likely to engage in peer relationships (52.6% vs 26.3%, NS) and support groups (26.3% vs 17.5%) than what they anticipated would be of value to them. See Figure 2 for more detailed comparisons of perceived beneficial resources vs those reportedly used by Group 1. Patients engaged in recovery services when they were available, even if the same patients previously considered such opportunities not worthwhile (see Group 1's perceptions versus actual use in Fig. 2).

**Table 3:**
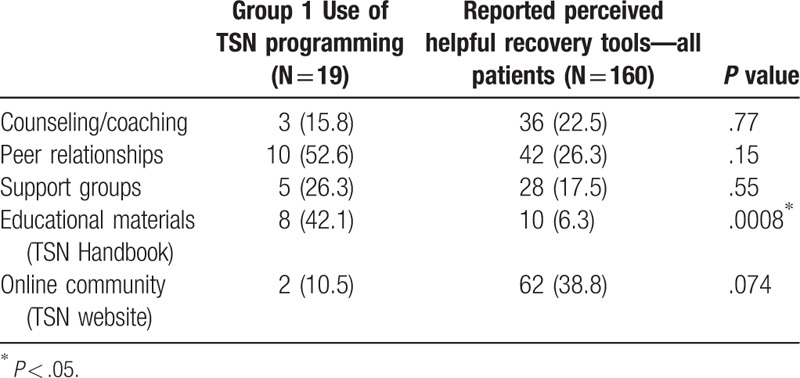
Reported use of resources versus perception of beneficial tools by nonexposed control groups.

### 3.7 TSN satisfaction

Patients who recalled use of recovery services were highly satisfied with these services, mean 4.42 (SD = 0.81). Group 1 patients with TSN exposure during their hospital stay similarly rated satisfaction with hospital care and self-efficacy when compared based on recalled use of TSN resources (Table 4).

**Table 4:**
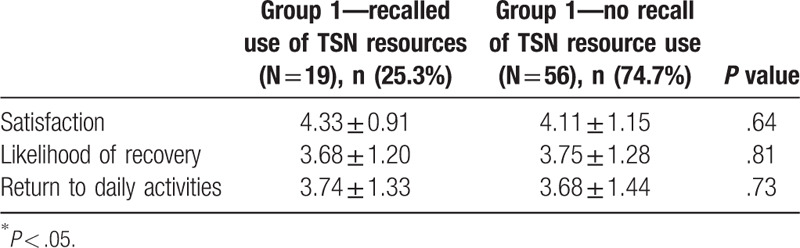
Impact of recall of services on survey responses.

## 4 Discussion

The results of our study indicate that the intervention of TSN programming was beneficial to trauma patients at our institution. Patients exposed to TSN programming (Group 1) had more self-efficacy with regard to overall recovery and return to daily activities. Recall of TSN resources was low. Only 25% of patients with prospectively recorded exposure to TSN programming reported using resources. This result may be limited by inaccurate memory of hospital course or recall bias. Self-reporting has been shown to be variable across patient populations.^[20–22]^ In a study of 101 active duty military personnel, recall of specific injuries was low, indicating some concern with self-reported data in traumatically injured populations with musculoskeletal injuries.^[23]^ Recall may be limited by pain, narcotic medication, fatigue, or severity of systemic injury. However, overall satisfaction with the program was high, indicated by an average of 4.42 on a Likert scale from 0 to 5.

Prior to the development of the TSN, there were limited comparable interventions in other medical fields and essentially no available recovery services for trauma populations. This is concerning, given the high demand for these services directed toward recovery in trauma patients. Mental illness is common among this cohort, with rates reaching as high as 45%,^[1–7]^ including 39% in a prior study within our hospital.^[6]^ Among trauma patients, depression appears to predominate, with other prevalent psychiatric illnesses being anxiety-related disorders, posttraumatic stress disorder (PTSD), and schizophrenia.^[2–4,6]^ Access to resources devoted to emotional and psychological recovery after traumatic injury may help to alleviate this burden, though further study is necessitated to ascertain potential benefits of TSN on mental illness.

The high prevalence of underlying mental illnesses is troubling for a number of reasons. Primarily, mental illness and substance abuse are key risk factors for subsequent recidivism, with rates reaching as high as 19% for patients treated for high-energy fractures.^[2,7,10]^ Mental illness has likewise been linked with higher complication rates, greater perception of pain, and poor functional outcomes.^[3,6]^ Several additional studies have indicated that worse functional outcomes scores are associated with deteriorating mental health, inadequate coping skills, and low social support.^[10,24–27]^ In a consistent study of 463 orthopaedic trauma patients, O’Toole et al^[28]^ found that mental illness and heightened pain were associated with lower patient satisfaction following treatment. Psychological status likewise impacts self-efficacy, a component thought crucial to optimal recovery after injury.^[24]^ Accordingly, in a cohort of patients with knee or hip arthroplasty, 6-week postoperative self-efficacy scores were correlated with long-term outcomes and function.^[29]^

Poor coping skills and other unconstructive cognitions following injury have also been implicated in increased pain and disability after musculoskeletal injury.^[30–32]^ Subsequently, in a cohort of patients treated operatively for one or more fractures, catastrophic thinking foreshadowed pain at rest and during daily activities, while pain anxiety was the sole predictor of disability following injury.^[30]^ Patient expectations also play a role in perception of pain,^[33]^ and therefore influence satisfaction with both hospital course and treating physicians.^[34]^ Self-reported likelihood to recover after trauma has been shown to predict higher patient ratings of satisfaction with their orthopaedic care.^[34]^

There have been few interventions in trauma populations aimed at addressing these concerns. Holman et al^[35]^ found in a cohort of trauma patients that proper counseling about opioid pain medications was associated with patients being more likely to follow recommendations and to reduce narcotic consumption in the anticipated time frame. This indicates a direct benefit from intervening to address concerns including mental health and substance use. Greden et al^[36]^ observed that instituting a peer-to-peer program in veteran populations led participants to seek out more formal medical services, demonstrating that such programs can help combat unmet needs of persons following trauma.

In a single-center prospective study of 251 patients (50% treated prior to TSN implementation and 50% treated following TSN implementation), Castillo et al showed similar impact as at our own institution. In this study, use of TSN resources also varied, from 3% of patients attending NextSteps classes to 27% receiving the TSN handbook. Use of services was higher among our respondents, with 26.3% using family classes or support groups and 42.1% receiving and using the TSN handbook.^[37]^ In the Castillo et al study, 47% of participants with follow-up data used at least 1 TSN resource. This is nearly identical to participation rates in our study, with 19 of 41 respondents (46%) recalling that they utilized TSN recovery tools. In the future, the authors would like to assess resources that patients both prefer and frequently use depending on demographics including age, gender, and social background. Successful implementation of recovery resources likely requires tailoring available services to the demographics of particular trauma centers, as this may differ considerably across urban and rural areas. Overall, satisfaction in both studies was high. Castillo et al found that 50% or more persons rated their satisfaction a minimum a 7 of 10 for support groups, TSN website, NextSteps, and peer advising. Although we did not assess satisfaction within individual subcategories, our overall satisfaction was high, with 58.3% of patients reporting 100% satisfaction.

If results are promising, why are there so few accessible resources? Primarily, nonpsychiatric healthcare providers often overlook mental health issues, thereby failing to address the psychosocial aspects which heavily influence recovery.^[6,38]^ Bradford et al^[13]^ reported that 10% of programs, only 3 trauma centers nationwide, initially trained in TSN programming fully implemented the program thereafter. Lack of both time and dedicated funding were the largest barriers to implementation, with other concerns being interdepartmental communication problems and lack of institutional support.^[13]^ These are no small issues, as it requires devotion from clinicians of all departments and hospital administration alike for TSN programming to be a success. A key overlooked component may be the development of relationships across disciplines and collaboration with physiatrists, counseling psychologists, and social workers to streamline enactment of a hospital TSN.

Patient perceptions of beneficial resources differed greatly with regards to actual use of available TSN tools. Group 1, which was exposed to TSN services, reported greater use of peer relationships, support groups, and educational materials than patients perceived to be helpful. Counseling and online resources were also used less often by Group 1 compared to perceptions of all groups. These findings are not unusual. Weighing perceived benefits and costs has been evidenced to sway patient-decision-making in numerous research scenarios including social, clinical, and biomedical studies.^[39–42]^ Accordingly, we assume that each patient that has TSN services made available to them most likely assesses the perceived benefits and costs to utilizing these resources. It is alarming however that barriers to individuals seeking mental health services have been widely reported to include perceived lack of need and ineffectiveness of treatment.^[43–46]^ These perceptions persist despite described benefits including minimizing stress and bolstering self-care routines gained from utilizing support groups, a key tool offered via TSN programming.^[47]^ In the future our team would like to investigate thoughts and perceptions of the TSN and how these may change over time, after patients leave the hospital and attempt to reengage with daily life activities.

The benefit of this study is its evaluation of the use and impact of a prospectively evolving recovery program, in the very first months of its implementation. Notably, despite the newness and limited experience of our program, we observed positive effects on self-efficacy. These types of services are fundamentally unavailable to most trauma populations worldwide. Our initial experience may aid other trauma centers in similar strategic initiatives and in related business planning regarding program impact. Limitations to this study include the small sample size and low response rate to mailed surveys. Furthermore, the survey was not validated. This study also assessed persons initially exposed to TSN programming in 2013. We did not determine the number of patients who declined services, although in our experience this almost never occurs. Program growth at our facility has greatly enhanced exposure to resources, as the program is now much more established. This initial data allows for important assessment of program expansion over time. Another significant limitation is the retrospective nature of the study. Patients were surveyed on average 8.2 months after surgical intervention and hospital stay. Not only may this shift the recall of impact over time, but it also allows patients who have recovered to more positively rate their satisfaction and self-efficacy. We suspect that this may be more of an issue for Group 3, which was a pre-TSN establishment cohort and therefore had a longer time to follow-up. There is no record of preinjury or subsequent mental illness for these patients, but we posit that the frequency of mental illness in these 3 groups of patients was likely similar. Going forward, we plan to assess the impact of TSN services on the prevalence of psychiatric illness following trauma to determine possible mitigation of postinjury depression, anxiety, or PTSD.

We conclude that patients were overall highly satisfied with their care, with no differences among groups. Additionally, our data indicate that TSN programming that addresses several key concerns following trauma, including self-management, peer support, information access, and online networking, was beneficial. These patients conveyed more self-efficacy regarding likelihood of recovery and return to daily activities. Given self-efficacy's impact on the recovery process, these patients were likely better equipped to cope with the lifestyle changes and pain that accompany traumatic injury. Finally, patients were highly satisfied with exposure to TSN resources. Trauma institutions and providers who implement similar programs at their institutions may enhance the recovery of their patients; further study in this area may elucidate additional efficacy of these types of services.
